# Baicalin promotes the sensitivity of NSCLC to cisplatin by regulating ferritinophagy and macrophage immunity through the KEAP1-NRF2/HO-1 pathway

**DOI:** 10.1186/s40001-024-01930-4

**Published:** 2024-07-26

**Authors:** Yang Chen, Shujun Bao, Zhongzhao Wang, Zheng Fang, Hao Tang

**Affiliations:** Department of Respiratory and Critical Care Medicine, Changzheng Hospital, Naval Medical University, Shanghai, 200003 China

**Keywords:** Baicalin (BA), KEAP1-NRF2/HO-1, Non-small cell lung cancer (NSCLC), Cisplatin (DDP)

## Abstract

**Background:**

Cisplatin (DDP) chemotherapy is commonly used in therapy for non-small cell lung cancer (NSCLC), but increased drug resistance has become a huge obstacle. Baicalin (BA) contributed to the sensitivity of NSCLC to DDP. Here, we aimed to further probe the pathophysiological mechanisms of BA in NSCLC.

**Methods:**

A549 and A549/DDP cells and xenograft mice were treated with BA and DDP. Xenograft mice were treated additionally with the NRF2 inducer (Bardoxolone methyl, BM) and KEAP1 knockdown. The levels of ferritinophagy-related proteins and biomarkers were determined. The autophagosomes were observed. M1 macrophage polarization and the contents of related indicators were analyzed. The involvement of KEAP1/NRF2/HO-1 was determined.

**Results:**

BA inhibited cell development, and the effect of BA and DDP on cell development was additive. The abundance of ferritinophagy-related proteins and the number of autophagosomes were induced by BA. BA also promoted the transition of GSH to GSSH. BA favored M1 macrophage polarization and affected the expression of related proteins. When BA and DDP combined, these molecular phenomena were further exacerbated. BA induced accumulation of KEAP1 and reduction of NRF2 and HO-1. However, BM and KEAP1 knockdown disrupted the synergistic effects of BA and DDP on inhibiting NSCLC growth. BM and KEAP1 knockdown reversed DDP and BA-promoted protein expression activity and M1 macrophage polarization.

**Conclusion:**

Our findings suggest that BA is involved in ferritinophagy and macrophage immunity through the KEAP1-NRF2/HO-1 axis, thereby improving the DDP sensitivity in NSCLC, which could provide new candidates for treatment strategies.

## Introduction

Lung cancer is reported to be the most common malignant tumor worldwide [1]. Non-small cell lung carcinoma (NSCLC) accounts for about > 80% of types of lung cancer, and its incidence is increasing yearly [2]. Currently, chemotherapy combined with new drugs occupies the mainstream position in the therapy for NSCLC. Among them, cisplatin (DDP)-based chemotherapy is the most common and effective treatment option in clinical practice [3]. However, due to enhanced drug resistance, the efficacy of DDP is limited, thereby resulting in greatly increased relapse and mortality in patients [4]. Therefore, there is an urgent need to explain the potential regulatory network involved in DDP resistance, which will help to improve the therapeutic efficacy of DDP.

Studies reported that large amounts of reactive oxygen species (ROS) accumulate in cancer cells to meet their demands for rapid growth and reconnection of metabolic networks due to abnormal metabolism and signal transduction [5]. If the concentration of ROS is excessively high, exacerbated oxidative stress promotes cell death and damage, thereby reducing the viability of cancer cells [6]. Nuclear factor erythroid 2-related factor 2 (NRF2) is described as an evolutionarily highly conserved transcription factor that targets transcriptional activation of oxidative stress-related genes, ultimately enhancing antioxidant capacity [7]. The activity of NRF2 is negatively regulated by kelch-like ECH-associated protein 1 (KEAP1) [8]. The KEAP1-NRF2 pathway is widely recognized as a promising pharmacological target for the treatment of NSCLC [9]. It has been reported that inhibition of NRF2 can promote autophagy to increase DDP chemosensitivity in NSCLC cells [10]. Autophagy was found to cause degradation of ferritin, which can lead to increased ROS levels, termed ferritinophagy [11]. Notably, NRF2 regulates the expression of many ferroptosis-related proteins and enzymes, including heme oxygenase 1 (HO-1), to reduce lipid peroxidation and ferroptosis [12, 13]. However, whether the KEAP1-NRF2/HO-1 pathway can regulate DDP resistance through ferritinophagy remains to be further demonstrated.

The medicinal sites of *Scutellariae Radix* (*SR*) accumulate a large amount of flavonoids, which have been shown to have a clear protective effect against NSCLC [14]. Baicalin (BA), the main pharmacodynamic component in *SR*, was found to enhance chemosensitivity to DDP [15, 16]. Studies have shown that the main active component complex of *SR* (containing BA) significantly enhanced macrophage viability and promoted M1 macrophage polarization in NSCLC to exert antitumor effects [17]. In addition, BA has been reported to regulate the mRNA expression of key autophagy genes, thereby inducing autophagy in cancer cells [18]. Interestingly, the formulation with BA as the main pharmacological component exhibited a significant anti-oxidative stress effect, which involved the KEAP1-NRF2 pathway [19, 20]. However, little has been reported on the effect of BA on DDP resistance via KEAP1-NRF2/HO-1 signaling.

To sum up, we explored the function of BA in the chemotherapy for NSCLC, which will help to uncover the underlying regulatory network of DDP resistance and develop novel drugs.

## Methods

### Cell culture and treatment

NSCLC cell line A549 cells (AW-CCH011, Abiowell) and their DDP-resistant cells (A549/DDP cells) (CL-0519, Procell) were fostered in DMEM and F-12 K medium, respectively, which were both supplemented with 10% fetal bovine serum (FBS) and 1% penicillin–streptomycin. Cells were complemented with 8 µg/mL BA (N1778, ApexBio) as the BA group, and 4 µg/mL DDP (C489606, Aladdin) was added as the DDP group [15]. BA and DDP were added together as the DDP + BA group. The control group received no additional treatment. All cells were incubated for 24 h to carry out subsequent experiments.

The indirect co-culture system was used to observe the effect of BA on macrophages in NSCLC. 5 × 10^5^/mL mouse macrophages (RAW264.7 cell line, ZQ0098, Shanghai Zhongqiao Xinzhou Biotechnology Co., Ltd.) were loaded into the upper chamber. 2.5 × 10^5^/mL of A549 or A549/DDP cells after treatment were added into the lower chamber. Then, RAW264.7 cells and A549 or A549/DDP cells were co-cultured for 24 h to obtain co-cultured lung cancer cells.

### Animal models

Sixty-six healthy nude mice (4-week-old) were provided from Hunan SJA Laboratory Animal Co., Ltd. All animal experiments in this study were approved by the Medical Ethics Committee of Shanghai Changzheng Hospital (No. LLSP20220420).

To investigate the role of BA, nude mice were randomly divided into control, BA, DDP, BA + DDP, and BA + DDP + BM groups, with 3 mice in each group. Nude mice were injected subcutaneously with 100 μL of A549 or A549/DDP cells at a concentration of 2 × 10^6^ cells for tumor formation. After 7 days of tumor formation, the mice were treated. In the control group, mice received the solvent used for BA (distilled water) orally, the solvent used for DDP (0.9% normal saline) by intraperitoneal injection, and the solvent used for bardoxolone methyl (BM, corn oil) by intraperitoneal injection. Mice in the BA group received 100 mg/kg BA (HY-N0197, MCE) orally [21] and 0.9% normal saline and corn oil by intraperitoneal injection. Mice in the DDP group received 3 mg/kg DDP [22] and corn oil by intraperitoneal injection and distilled water orally. Mice in the DDP + BA group received 100 mg/kg BA orally and 3 mg/kg DDP and corn oil by intraperitoneal injection. Mice in the DDP + BA + BM group received 100 mg/kg BA orally and 3 mg/kg DDP and 10 mg/kg BM (218600-53-4, Abmole) [23] by intraperitoneal injection.

To investigate the involvement of the KEAP1-NRF2/HO-1 pathway, nude mice were randomly divided into control, sh-Keap1, BA + DDP, BA + DDP + sh-Keap1, DDP + DTX and BA + DDP + DTX groups, with 3 mice in each group. In the control and BA + DDP groups, nude mice were injected subcutaneously with 100 μL of A549 or A549/DDP cells transfected with sh-NC (2 × 10^6^) for tumor formation. Nude mice in the sh-Keap1 and BA + DDP + sh-Keap1 groups were injected subcutaneously with 100 μL of A549 or A549/DDP cells transfected with sh-Keap1 (2 × 10^6^) for tumor formation. Nude mice in the DDP + DTX and BA + DDP + DTX groups, nude mice were injected subcutaneously with 100 μL of A549 or A549/DDP cells (2 × 10^6^) to induce tumor formation. After 7 days of tumor formation, the mice were treated. Specifically, mice in the control group received orally distilled water (solvent for BA), intraperitoneal injection of 0.9% normal saline (solvent for DDP) and tail vein injection of 20% SBE-β-CD (solvent for DTX). Mice in the sh-Keap1 group did not receive any additional drugs. Mice in the BA + DDP group received 100 mg/kg BA orally, 3 mg/kg DDP by intraperitoneal injection and 20% SBE-β-CD by tail vein injection. Mice in the BA + DDP + sh-Keap1 group received 100 mg/kg BA orally and 3 mg/kg DDP by intraperitoneal injection. Mice in the DDP + DTX group received oral distilled water, intraperitoneal injection of 3 mg/kg DDP and tail vein injection of 5 mg/kg docetaxel (DTX) [24]. Mice in the BA + DDP + DTX group received oral 100 mg/kg BA, intraperitoneal injection of 3 mg/kg DDP and tail vein injection of 5 mg/kg DTX.

The operation was terminated on the 28th day, and the mouse and tumor weight were recorded within 28 days. Mice were sacrificed after intraperitoneal injection of 150 mg/kg pentobarbital sodium, and the tumors were weighed and collected for subsequent experiments.

### Cell counting kit-8 (CCK-8) assay

Cell viability of A549 and A549/DDP cells was detected by CCK8 assay. Briefly, cells at a 5 × 10^3^/well density were seeded in 96-well plates. 10 μL of CCK8 (NU679, DOJIMDO) was added for incubation. The absorbance was read at 450 nm using a microplate reader (MB-530, HEALES).

### Determination of cell cycle

A549 and A549/DDP cells were resuspended in pre-chilled PBS solution and fixed with 75% ethanol overnight at 4 °C. Ethanol was removed, and propidium iodide (MB2920, Meilunbio) was added for staining in the dark for 30 min. Finally, a flow cytometer (A00-1-1102, Beckman) was utilized to examine the distribution of cell cycle phases.

### Terminal deoxynucleotidyl transferase-mediated dUTP nick end labeling (TUNEL) assay

Apoptosis in A549 and A549/DDP cells was assessed based on a kit instruction (40306ES50, YEASEN). Cells were mixed with TUNEL solution to incubate for 1 h. Nuclei were subsequently stained. The positive cells were counted using a fluorescence microscope (BA410T, Motic).

### Transwell assay

The migratory and invasive abilities of A549 and A549/DDP cells were assessed utilizing transwell chambers (3428, Corning). Extra Matrigel (354262, BD) were applied for cell invasion assay. Complete medium containing 10% FBS was added to the lower chamber. Cells were trypsinized and resuspended in serum-free medium to a concentration of 2 × 10^6^ cells/mL. The cell plate was then placed at 37 °C for 48 h. Then, cells were stained with 0.1% crystal violet (AWC0333, Abiowell). Finally, cells were observed under a microscope, and the absorbances were measured at 550 nm after destaining.

### Quantitative real-time PCR (qRT-PCR)

Lung cancer cell supernatant was mixed with TRIzol (15596026, Thermo) to extract total RNA, followed by reverse transcription into cDNA using a cDNA synthesis kit (CW2569, CWBIO). The cDNA was amplified with the UltraSYBR Mixture (CW2601, CoWin Biosciences) and analyzed utilizing QuantStudio 1 Real-Time PCR (Thermo). 2^−ΔΔCt^ was utilized to calculate the relative expression of target genes with β-actin as a reference. The primer sequences are exhibited in Table 1.

**Table 1:** Primer sequence

Gene	F (5′-3′)	R (5′-3′)
KEAP1	CGTGGCTGTCCTCAATCGTCT	ATTGCTGTGATCATTCGCCACT
NRF2	CAACTCAGCACCTTATATCTCG	ACAAGGAAAACATTGCCATC
HO-1	CACACCCAGGCAGAGAATGCT	GGCTCTCCTTGTTGCGCTCA
SLC7A11	CTCCAGGTTATTCTATGTTGCGTCT	CAAAGGGTGCAAAACAATAACAGC
GPX4	CGCCTTTGCCGCCTACTGAAGC	AACCATGTGCCCGTCGATGTCC
β-actin	ACCCTGAAGTACCCCATCGAG	AGCACAGCCTGGATAGCAAC

### Western blot

The collected A549 and A549/DDP cells or mouse lung tissue were mixed well with RIPA lysate (AWB0136, Abiowell). The supernatant was obtained after centrifugation at 4 °C, and the sample concentration was determined using a BCA kit (AWB0104, Abiowell). Total protein was separated by electrophoresis and transferred to membranes. Membranes were mixed with primary antibodies at 4 °C, including KEAP1 (1:5000, 10503-2-AP, Proteintech), NRF2 (1:1000, 16396-1-AP, Proteintech), HO-1 (1:3000, 10701-1-AP, Proteintech), SLC7A11 (1:1000, 26864-1-AP, Proteintech), GPX4 (1:1000, 67763-1-Ig, Proteintech), SLC40A1 (1:1000, bs-4906R, Bioss), transferrin (1:1000, 17435-1-AP, Proteintech), LC3 (1:500, 18725-1-AP, Proteintech) and β-actin (1:5000, 66009-1-Ig, Proteintech). Then, HRP-labeled mouse antibody (1:5000, SA00001-1, Proteintech) or rabbit antibody (1:6000, SA00001-2, Proteintech) was added for incubation. Protein bands were visualized using ECL Plus ultra-sensitive luminescence solution (AWB0005, Abiowell).

### Immunofluorescence (IF) analysis

The expression of HO-1 (1:50, 10701-1-AP, Proteintech) and LC3 (1:100, 14600-1-AP, Proteintech) were analyzed by IF. Briefly, cells were incubated with 0.3% triton for 30 min after fixation. Cells were blocked with 0.5% BSA for 60 min and incubated with primary antibody overnight at 4 °C. Cells were then mixed with CoraLite488-conjugated Goat Anti-Rabbit IgG (H + L) (1:200, SA00013-2, Proteintech) for incubation. Nuclei were stained with DAPI. Finally, the green fluorescence was captured by microscopy.

### Transmission electron microscope (TEM) analysis

A549 and A549/DDP cells were resuspended in 2.5% glutaraldehyde (AWI0097, Abiowell) and 1% osmium tetroxide (18456, TED PELLA). Cells were sequentially dehydrated, embedded, sectioned, and stained. Finally, the autophagy situation in cells was observed by a microscope (JEM1400, JEOL).

### ROS assay

ROS levels were evaluated by a ROS kit (S0033S, Beyotime). 10 μmol/L DCFH-DA was added for co-incubation with cells at 37 °C for 20 min. The labeled cells were trypsinized, and the fluorescence intensity was measured by flow cytometry.

### Lipid peroxidation assay

Lipid peroxidation levels were detected by applying C11-BODIPY (D3861, Thermofisher). 5 μmol/L C11-BODIPY was added and then cells were incubated at 37 °C for 30 min. Fluorescence intensity was detected by flow cytometry after washing with PBS.

### Immunophenotyping

The tumors were ground and then centrifuged at 1500 rpm for 10 min to obtain cell pellets. Cells were resuspended in the erythrocyte lysate, and the supernatant was removed after centrifugation. After washing with PBS, F4/80-FITC (11-4801-82, eBioscience) and CD11c-PE (12-0114-82, eBioscience) probes were added for incubation in the dark, ultimately detecting the percentages of F4/80^+^ and CD11c^+^.

### Determination of biomarkers of ferritinophagy

The Fe^2+^ levels were detected using a microplate reader. The activities of reduced glutathione (GSH) and oxidized glutathione (GSSG) in lung cancer cells were assessed using the GSH assay kit (A006-2) and Toal glutathione/GSSG assay kit (A061-1, Nanjing Jiancheng Bioengineering Institute). All operations were performed based on the instructions.

### Enzyme-linked immunosorbent assay (ELISA)

The contents of VEGFA (KE00216, Proteintech), TNF-α (KE00068, Proteintech), TGF-β1 (KE00002, Proteintech), and iNOS (CSB-E08148h, Elabscience) were identified based on the operation of ELISA kit.

### Statistical analysis

Statistical analysis was performed by applying Graphpad Prism 8. Each value was presented as mean ± standard deviation. Differences between the two groups were monitored by *t-test*. One-way analysis of variance (ANOVA) was used to determine the differences more than the two groups. *P* < 0.05 was explained as statistically significant.

## Results

### BA promoted DDP-induced chemosensitivity of human lung cancer cell

**Fig. 1:**
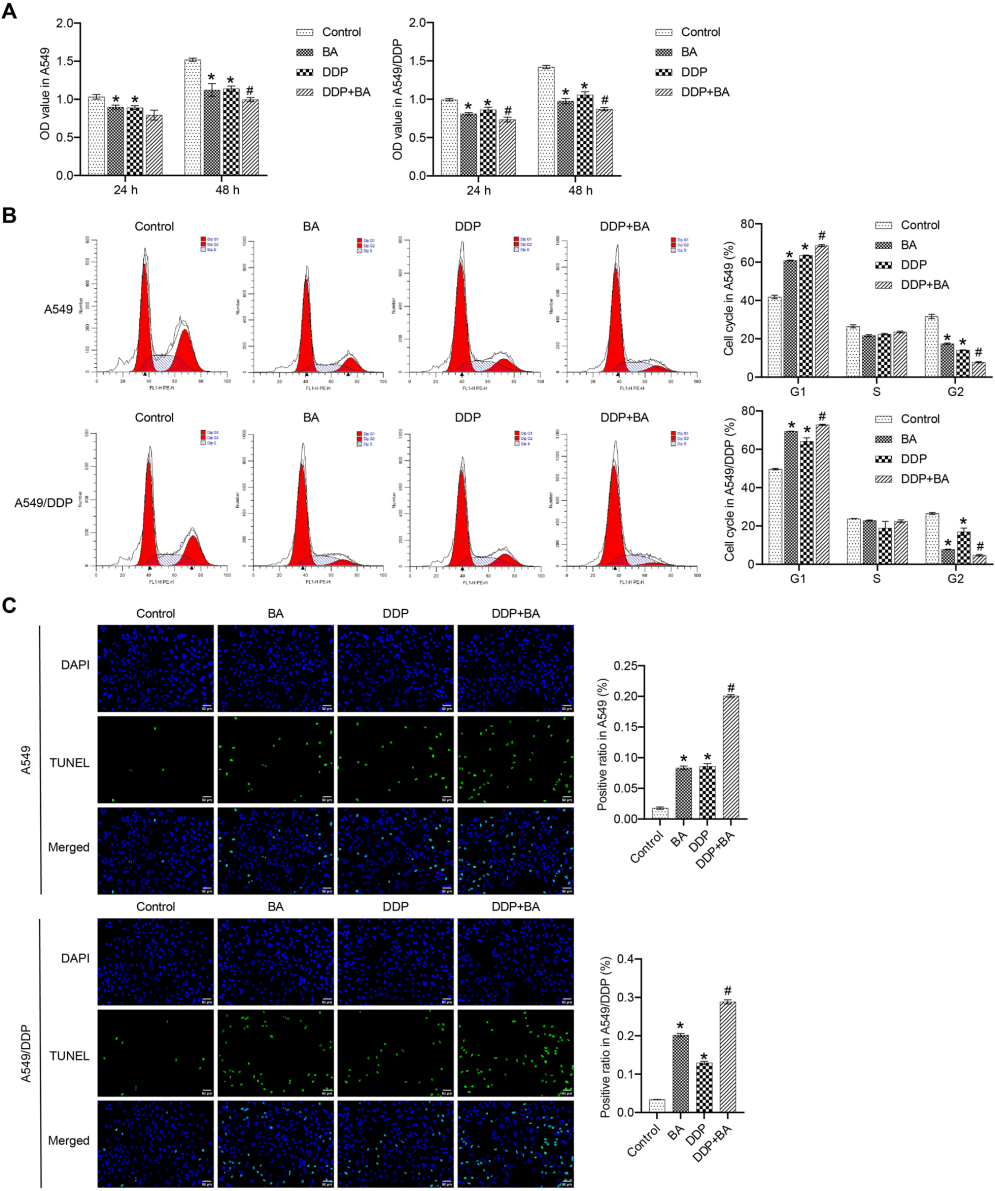

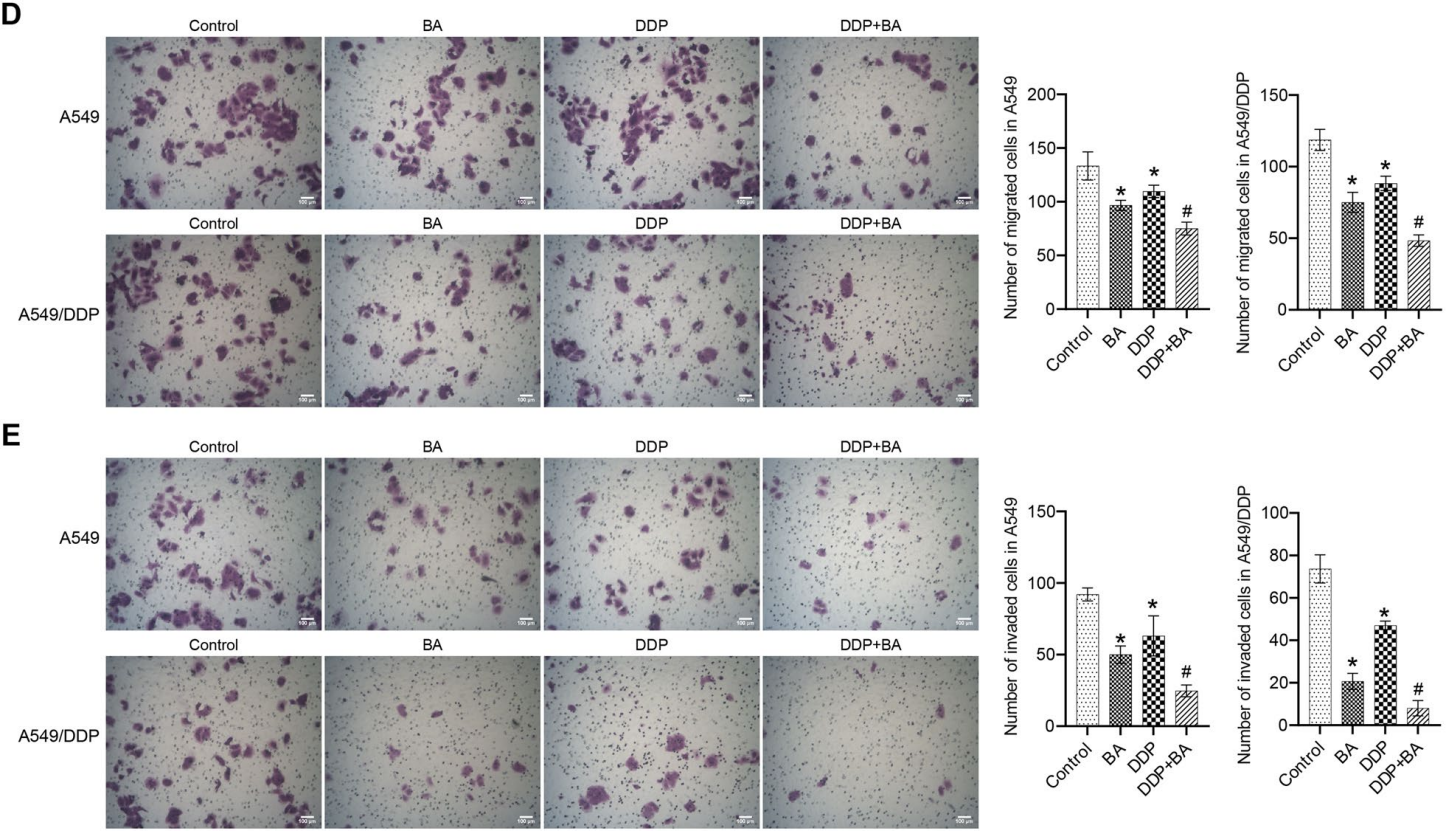
BA strengthened the susceptibility of A549 and A549/DDP cells to DDP. **A** Cell viability was estimated by CCK8 assay; **B** Flow cytometry was applied to assess the distribution of cell cycle phase; **C** The level of apoptosis was measured by TUNEL assay, scale bar = 50 μm; **D** Transwell assay was employed to analyze cell migration, scale bar = 100 μm. **E** Transwell assay was used to analyze cell invasion, scale bar = 100 μm. *P < 0.05 *vs* Control; #*P* < 0.05 *vs* DDP

We evaluated the effect of DDP and BA on human lung cancer cells. The results showed that DDP and BA caused a significant decrease in cell viability compared to the Control group, and the combination of DDP and BA further aggravated this phenomenon (Fig. 1A). In A549 and A549/DPP cells, DDP and BA greatly induced G1 cell cycle arrest while decreasing the percentage of cells in the G2 phase, and BA further enhanced the effect of DDP (Fig. 1B). DDP and BA increased the proportion of apoptosis in A549 and A549/DPP cells, and the highest level of apoptosis was observed in the DDP + BA group (Fig. 1C). Transwell experiments showed that DDP and BA reduced the migration and invasion of A549 and A549/DDP cells, and the combination of the two accelerated this trend (Fig. 1D and E). These results suggested that BA sensitizes A549 and A549/DDP cells to DDP, which is manifested in delayed cell cycle, enhanced cancer cell apoptosis, and reduced cancer cell viability, migration, and proliferation.

### BA activated KEAP1-NRF2/HO-1 pathway and accelerated autophagy

**Fig. 2:**
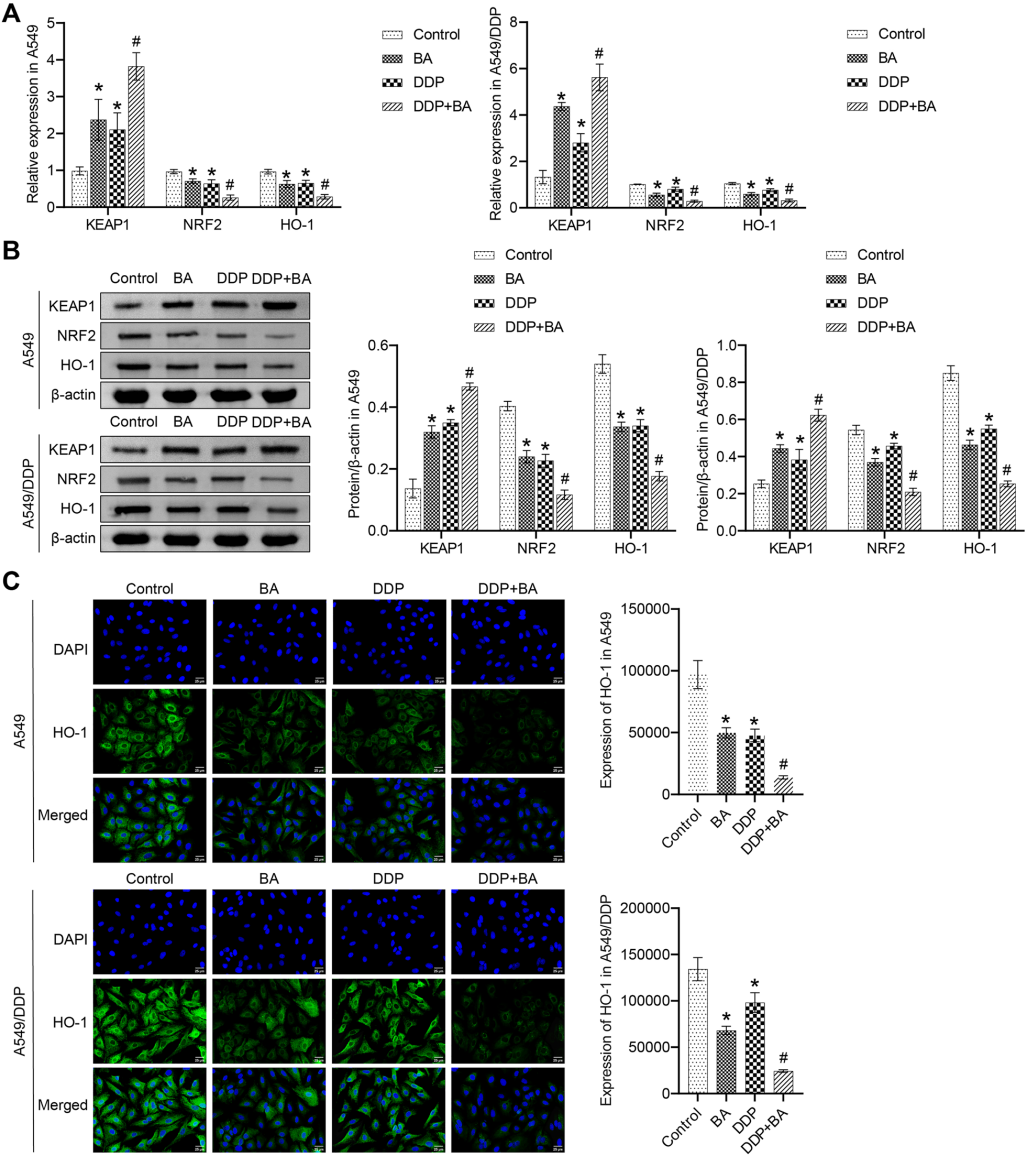

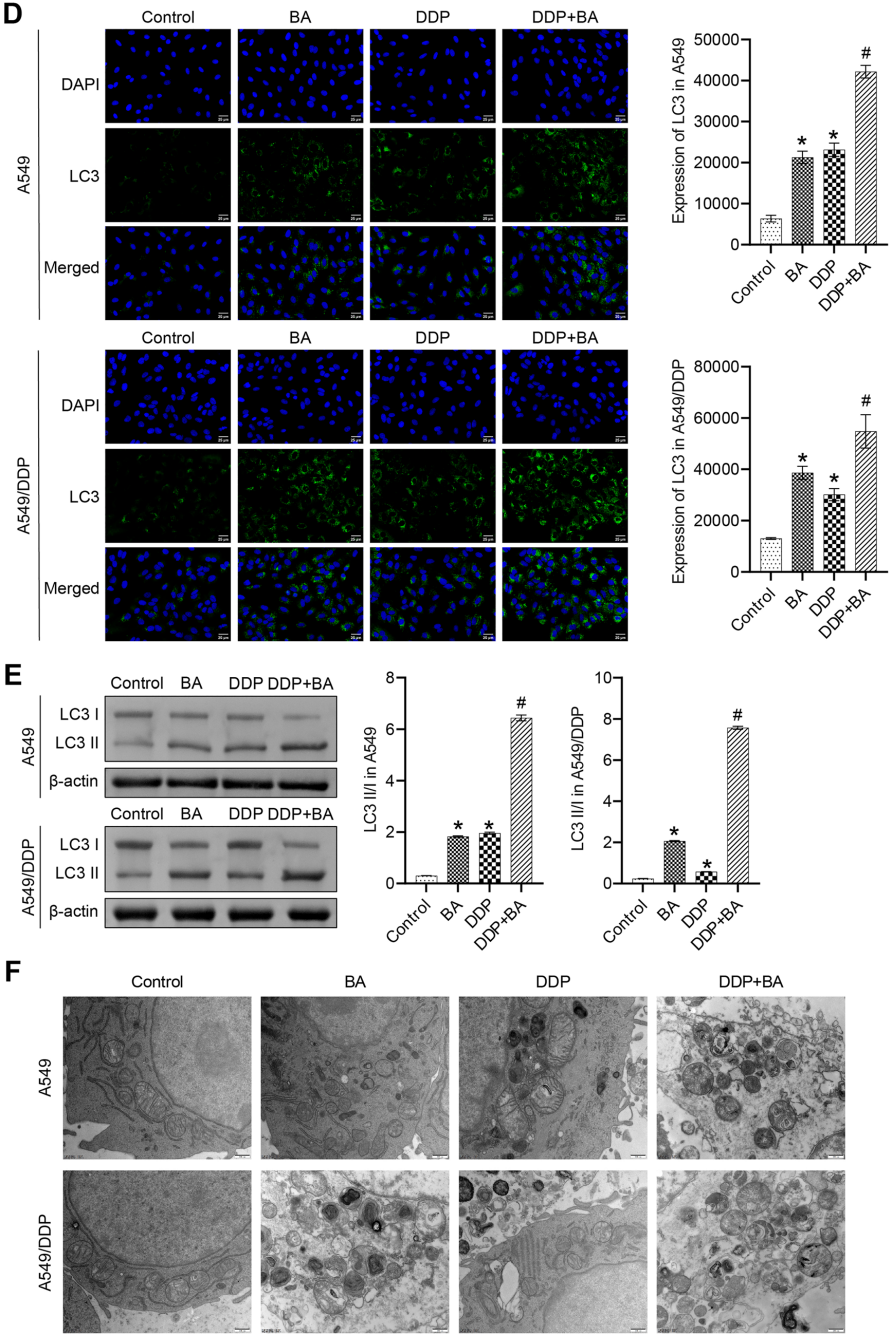
BA regulated DDP sensitivity through the KEAP1-NRF2/HO-1 pathway. **A** The relative levels of KEAP1, NRF2, and HO-1 in A549 and A549/DDP cells were examined by qRT-PCR; **B** Western blot was utilized to measure the protein abundance of KEAP1, NRF2, and HO-1; **C** IF assay was applied to analyze the activities of HO-1, scale bar = 25 μm; **D** The level of LC3 was assessed by IF, scale bar = 25 μm; **E** Western blot was used to measure the expression of LC3 II and I; **F** Cell autophagy was observed on a TEM, scale bar = 500 nm. *P < 0.05 *vs* Control; #*P* < 0.05 *vs* DDP

To investigate whether BA regulates KEAP1-NRF2/HO-1 signaling in A549 and A549/DDP cells, the accumulation of KEAP1, NRF2, and HO-1 wA measured. Separate BA or DDP significantly increased the accumulation of KEAP1 and inhibited the levels of NRF2 and HO-1, and the combination of the two further induced the expression trend of KEAP1, NRF2 and HO-1 (Fig. 2A and B). As shown in Fig. 2C, the results of IF further verified that the expression of HO-1 was regulated by BA and DDP. In addition, the activity of the autophagy-related protein LC3 was positively regulated by BA and DDP, and BA accelerated the DDP-induced accumulation of LC3 (Fig. 2D). BA or DDP alone increased the ratio of LC3 II/I compared to the control group, and the combination of the two further increased the ratio (Fig. 2E). The observation results displayed that the number of autophagosomes in the control group was less, and the organelle structure was relatively complete. The number of autophagosomes in the BA or DDP groups was higher than that in the control group, and a large number of autophagolysosomes were found in the DDP + BA group (Fig. 2F). Taken together, these data manifested that BA activated KEAP1-NRF2/HO-1 signaling and induced increased autophagy in vitro to improve DDP-enhanced anticancer effects.

### BA-induced ferritinophagy in A549 and A549/DDP cells

**Fig. 3:**
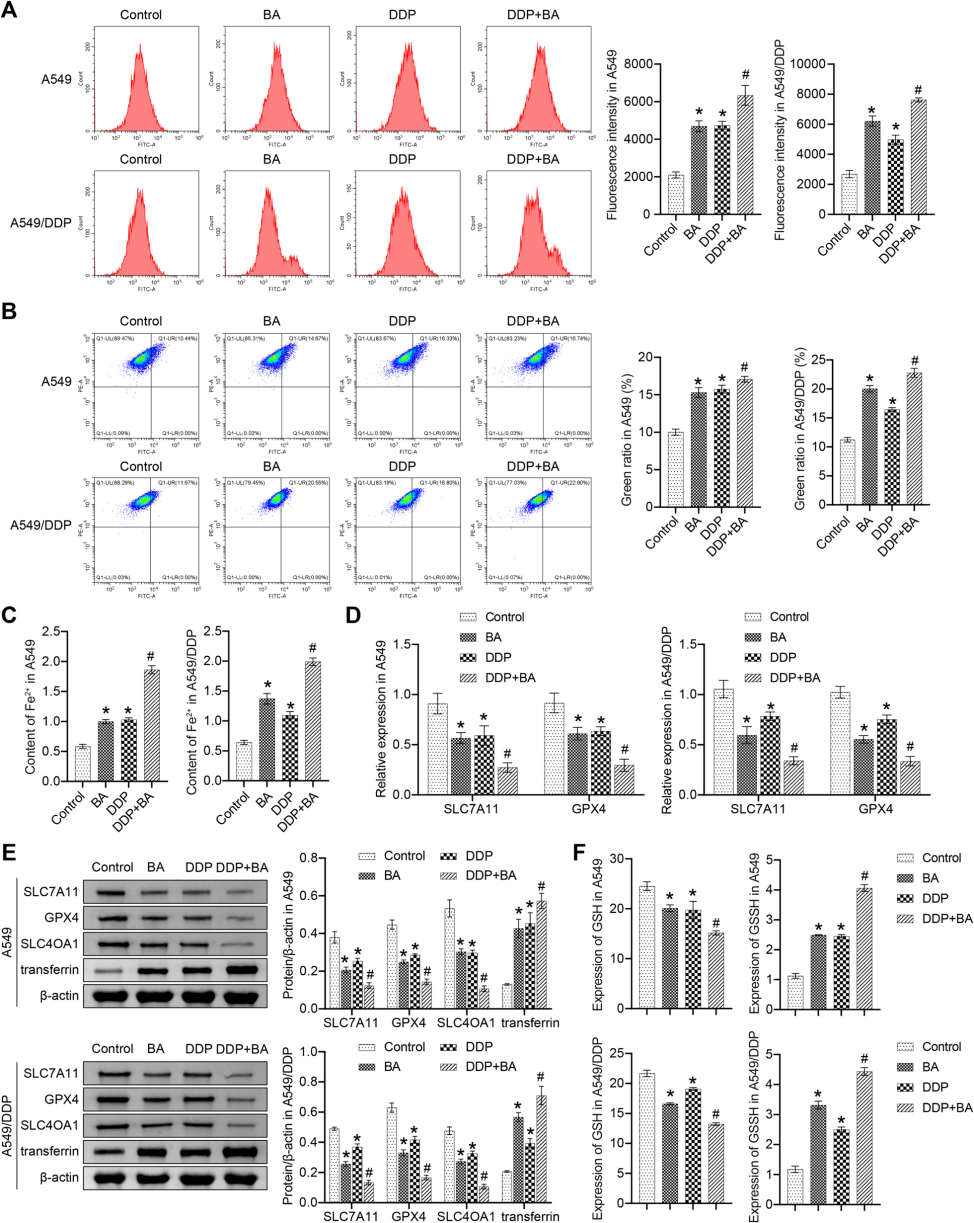
BA accelerated ferritinophagy in A549 and A549/DDP cells. **A** ROS levels were assessed by flow cytometry; **B** Changes in lipid peroxidation were detected; **C** The activity of Fe^2+^ was examined; **D** qRT-PCR was used to measure the relative mRNA levels of SLC7A11, and GPX4 in cells; **E** The relative abundances of SLC7A11, GPX4, SLC40A1, and transferrin were determined by western blot; **F** GSH and GSSH levels were calculated. *P < 0.05 *vs* Control; #*P* < 0.05 *vs* DDP

Previous reports demonstrated that excessive activation of autophagy promotes ferroptosis [25]. Ferroptosis involves the accumulation of ROS and lipid peroxidation [26]. Flow cytometry results illustrated that BA and DDP were beneficial to the increase of ROS and lipid peroxidation in A549 and A549/DDP cells, and the combination of the two significantly further promoted ROS production and lipid peroxidation (Fig. 3A and B). The content of Fe^2+^ was greatly increased in the BA and DDP groups, and the abundance of Fe^2+^ was further increased in the DDP + BA group (Fig. 3C). The contents of solute carrier family 7 member 11 (SLC7A11), glutathione peroxidase 4 (GPX4), and SLC40A1 were decreased by BA and DDP, and transferrin increased (Fig. 3D and E). BA further increased DDP-enhanced expression activity of these markers. Changes in the levels of GSH and GSSG can be used to measure ferritinophagy in cells [27]. As shown in Fig. 3F, BA and DDP induced a decrease in GSH levels and an increase in GSSH levels, and the combination of the two accelerated this trend. In short, these findings illustrated that BA promoted ferritinophagy in A549 and A549/DDP cells.

### BA contributed to anticancer immune responses

**Fig. 4:**
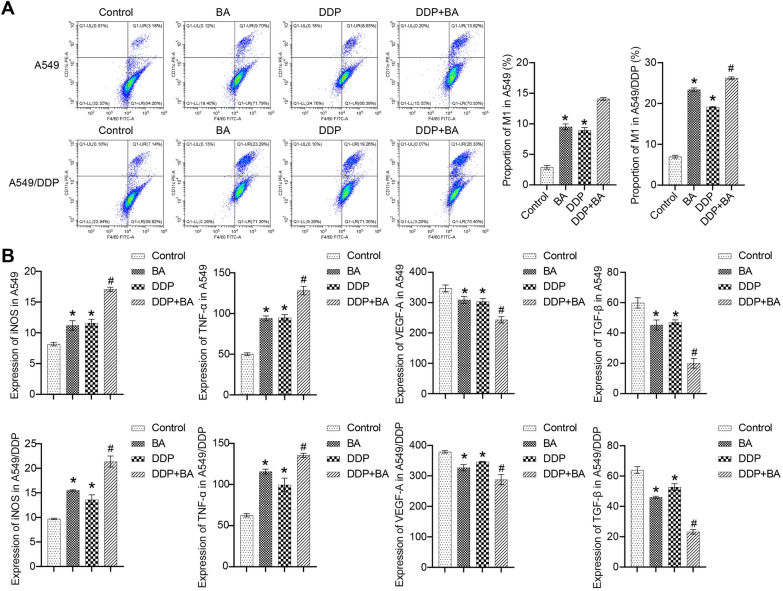
BA promoted M1 macrophage polarization. **A** The proportion of M1 macrophages (F4/80^+^ and CD11c^+^) was evaluated by flow cytometry; **B** The expressions of iNOS, TNF-α, VEGF-A, and TGF-β were examined by ELISA. *P < 0.05 *vs* Control; #*P* < 0.05 *vs* DDP

Substantial evidence reveals that the complex tumor microenvironment (TME) is critical for the treatment of NSCLC [28]. As shown in Fig. 4A, the ratio of F4/80^+^ to CD11c^+^ was markedly up-regulated by BA and DDP in A549 and A549/DDP cells, and the combination of the two further increased the ratio. BA and DDP increased secretion of M1-related cytokines, including inducible nitric oxide synthase (iNOS) and tumor necrosis factor-α (TNF-α), and decreased secretion of M2-related cytokines, including vascular endothelial growth factor-A (VEGF-A) and transforming growth factor-β (TGF-β), and BA further enhanced the level changes of DDP-regulated cytokines (Fig. 4B). These data suggested that BA contributed to M1 macrophage polarization in lung cancer cells to enhance DDP sensitivity.

### BM reversed BA-induced effects in mice

**Fig. 5:**
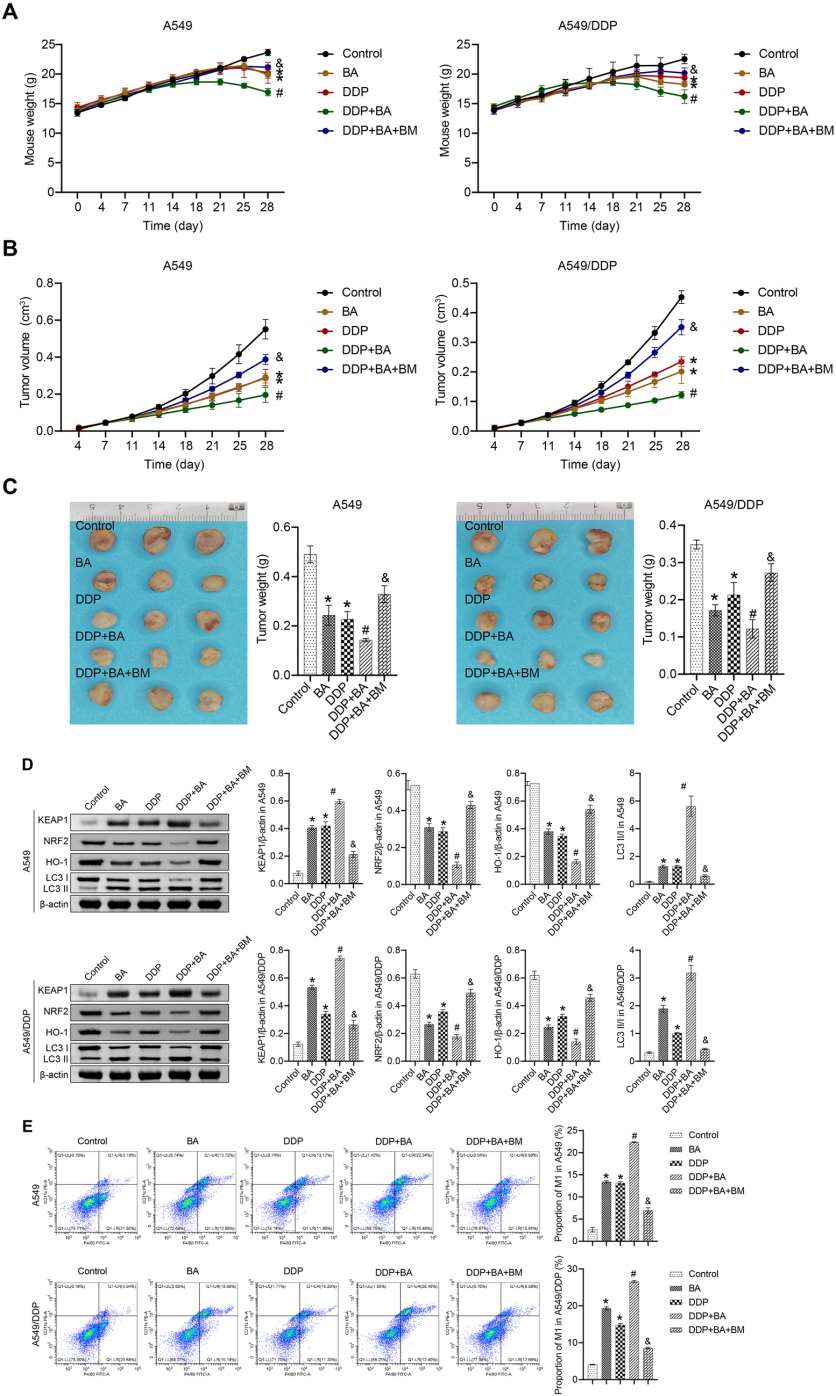
BA modulated the sensitivity of NSCLC to DDP via the KEAP1-NRF2/HO-1 pathway in vivo. **A** Mouse growth curve in different groups; **B** Tumor volume was estimated twice a week; **C** Tumors were weighed on the 28th day; **D** The abundance of KEAP1, NRF2, HO-1, and LC3 was detected by western blot; **E** Flow cytometry was applied to measure the ratio of M1 macrophages (F4/80^+^ and CD11c^+^). *P < 0.05 *vs* Control; #*P* < 0.05 *vs* DDP; &*P* < 0.05 *vs* DDP + BA

The KEAP1-NRF2/HO-1 pathway has been reported to be involved in ferroptosis in vitro and in vivo [29]. We administered an NRF2 inducer (BM) to explore whether BA modulates DDP sensitivity through KEAP1-NRF2/HO-1 signaling in vivo [23]. We developed xenograft mouse models of A549 and A549/DDP cells. Compared with the Control group, the BA and DDP groups showed a self-evident inhibitory effect on the NSCLC tumor in mice, and the combination of the two further improved NSCLC in the mice. Interestingly, the DDP + BA + BM group exhibited similar tumor volume and weight to the Control group, disrupting the therapeutic effects of BA and DDP (Fig. 5A, B and C). We further analyzed the potential regulatory mechanisms in vivo. The regulation of KEAP1, NRF2, HO-1, and LC3 II/I levels by BA and DDP was consistent with the cellular experiments, and BM clearly reversed the functions of BA and DDP (Fig. 5D). Then, we examined the proportion of M1 macrophages in the tumor. Compared with the Control group, BA and DDP induced M1 macrophage polarization, and the DDP + BA group exhibited a higher proportion of M1 macrophages, while BM sharply suppressed the effects of BA and DDP (Fig. 5E). These results confirmed in vivo that BA could promote the sensitivity of NSCLC to DDP by triggering autophagy and M1 macrophage polarization through the KEAP1-NRF2/HO-1 pathway.

### KEAP1 knockdown disrupted the protective effects of DDP and BA

**Fig. 6:**
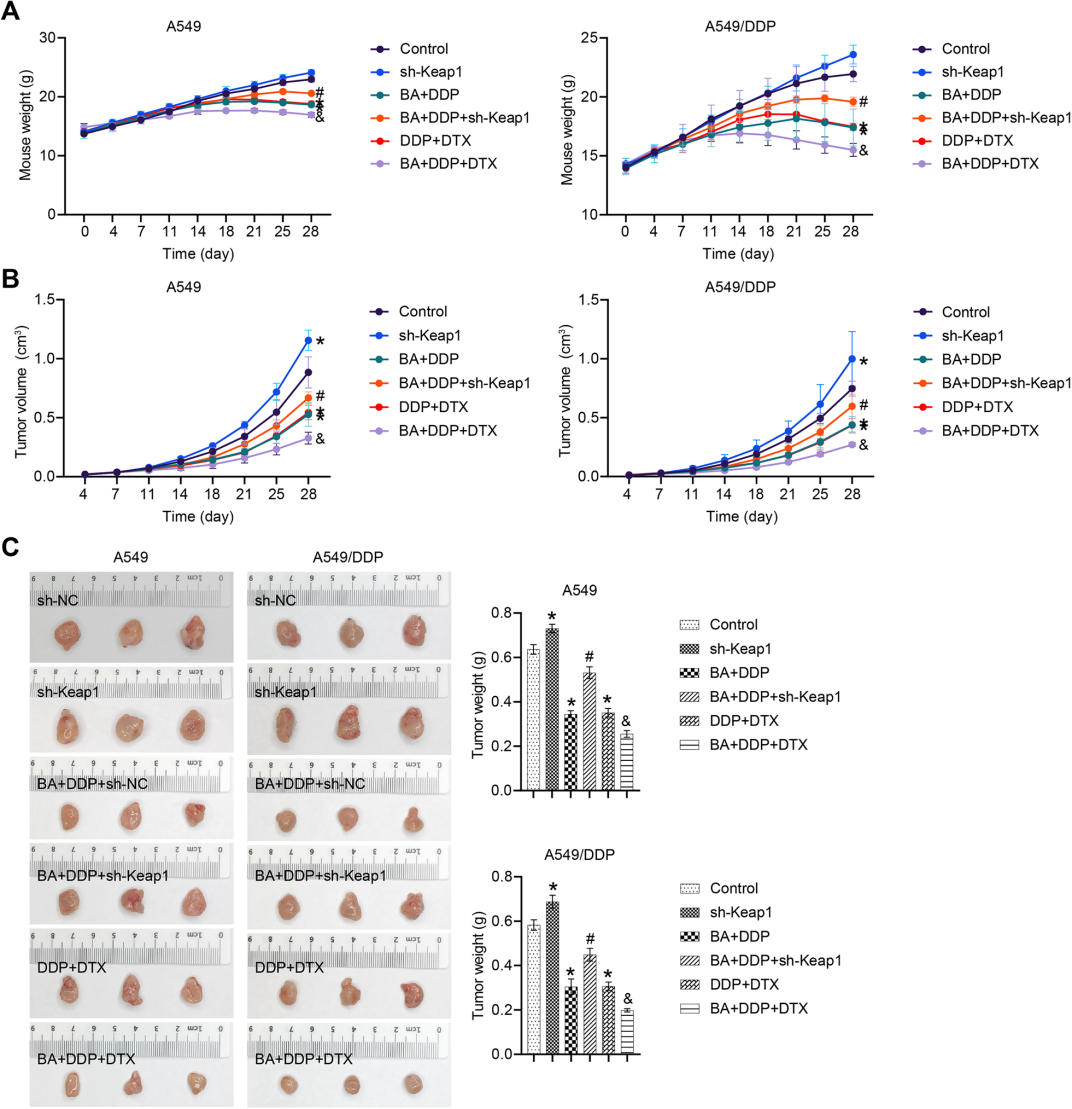

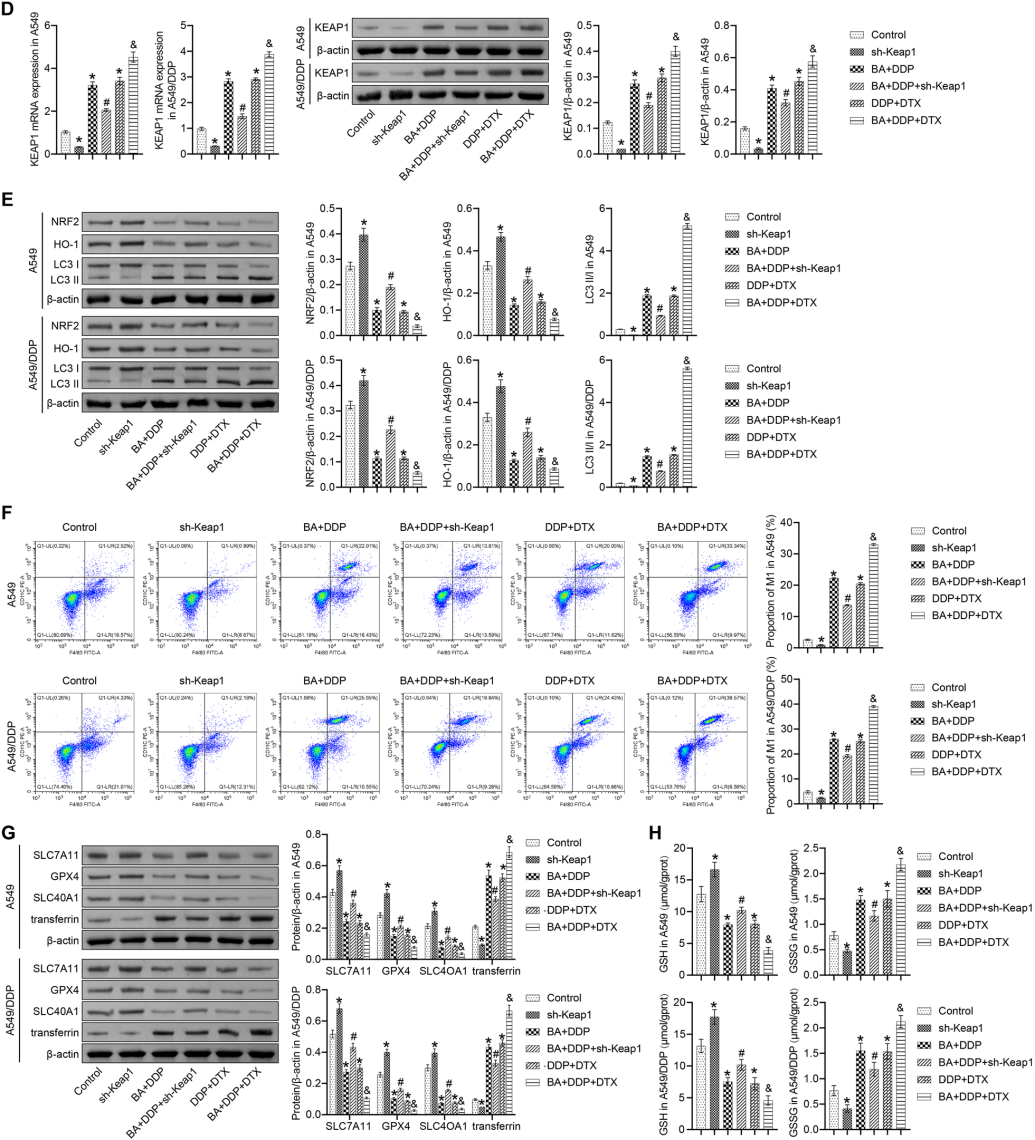
The KEAP1-NRF2/HO-1 pathway was involved in BA-mediated sensitivity of NSCLC to DDP in vivo. **A** Mouse growth curve in different groups; **B** Tumor volume was estimated twice a week; **C** Tumors were weighed on the 28th day; **D** The expression of KEAP1 was determined by qRT-PCR and western blot; **E** The abundance of NRF2, HO-1, and LC3 was detected by western blot; **F** Flow cytometry was applied to measure the ratio of M1 macrophages (F4/80^+^ and CD11c^+^). **G** The protein levels of SLC7A11, GPX4, SLC40A1, and transferrin were determined by western blot; **H** GSH and GSSH levels were determined. *P < 0.05 *vs* Control; #*P* < 0.05 *vs* BA + DDP; &*P* < 0.05 *vs* DDP + DTX

The involvement of the KEAP1-NRF2/HO-1 pathway in the BA-mediated sensitivity of NSCLC to DDP was further elucidated. The results showed that compared to the control group, sh-Keap1-intervened mice exhibited increased tumor volume and weight, while the BA/DDP combination and the DDP/DTX combination exerted protective effects. Compared to the BA + DDP group, sh-Keap1 intervention disrupted the therapeutic effects of BA/DDP, resulting in decreased body weight and increased tumor volume and weight. Compared to the DDP + DTX group, BA further enhanced the protective effect of the DDP/DTX combination (Fig. 6A, B and C). Compared to the control group, sh-Keap1 resulted in downregulation of KEAP1 and LC3 II/I expression and upregulation of NRF2 and HO-1 expression, while the BA/DDP combination and the DDP/DTX combination exerted opposite effects. Compared to the BA + DDP group, sh-Keap1 reduced the expression of KEAP1 and LC3 II/I and promoted the expression of NRF2 and HO-1. Compared to the DDP + DTX group, BA further increased the expression of KEAP1 and LC3 II/I and decreased the expression of NRF2 and HO-1 (Fig. 6D and E). Mice injected with sh-Keap1-transfected cells showed a decreased proportion of M1 macrophages in the tumors compared to the BA + DDP group. The DDP/DTX combination increased the proportion of M1 macrophages in the tumors compared to the control group and BA amplified this effect (Fig. 6F). Furthermore, the BA + DDP + sh-Keap1 group showed increased expression of SLC7A11, GPX4 and SLC40A1 and decreased expression of transferrin compared to the BA + DDP group. The DDP/DTX combination attenuated the expression of SLC7A11, GPX4 and SLC40A1 and promoted the expression of transferrin and BA further amplified these trends (Fig. 6G). Compared with the control group, sh-Keap1 induced an increase in GSH levels and a decrease in GSSH levels, while the BA/DDP combination and the DDP/DTX combination had opposite effects. sh-Keap1 weakened the effect of the BA/DDP combination, and BA enhanced the effect of the DDP/DTX combination (Fig. 6H). Importantly, nude mice in the BA + DTX group showed similar results to the DDP + DTX group (Fig. 6). These results further confirmed in vivo that BA regulated ferritinophagy and M1 macrophage polarization through the KEAP1-NRF2/HO-1 pathway.

## Discussion

*SR* is a traditional Chinese medicinal material widely used as an adjuvant for chemotherapy in clinical practice [30]. Currently, more than 60 flavonoids have been isolated and identified in *SR*, and these compounds show strong antioxidant activities [31]. Among them, wogonin, baicalein and BA are the main active components of *SR*, which have excellent antitumor effects against multiple types of cancer, such as glioblastoma, colorectal cancer, prostate cancer, gastric cancer, etc. [32]. Evidence suggests that *SR* can be applied to prevent and treat NSCLC [14, 33]. However, few reports have focused on the function of *SR* in the sensitivity of NSCLC to cisplatin chemotherapy. In our study, we reported that BA decreased cell viability, reduced cell migration and invasion, accelerated apoptosis and cell cycle arrest in NSCLC parental cell line (A549) and cisplatin-resistant cell line (A549/DDP), ultimately modulating chemotherapy response in NSCLC. The single application of BA and DDP contributed to inhibiting NSCLC growth, and the confederate application of the two further enhances the efficacy of BA and DDP. DTX, as an effective chemotherapeutic agent, exhibits excellent antitumor and targeted activities in NSCLC [34, 35]. Combination therapy of DTX and DDP has shown promising treatment outcomes [36, 37]. BA could improve current adjuvant therapies such as the DDP/DTX combination in NSCLC.

The KEAP1-NRF2/HO-1 pathway plays a significant role in regulating cellular redox status. In unstressed conditions, NRF2 is located in the cytoplasm, and KEAP1 promotes NRF2 ubiquitination and proteasomal degradation to maintain NRF2 levels at a very low concentration. Once the accumulation of ROS in cells is abnormal, NRF2 is released from KEAP1 and translocated into the nucleus, thereby activating the transcription of antioxidant genes, including HO-1 [38]. We reported that BA promoted the accumulation of KEAP1 and inhibited NRF2 and HO-1 in vitro and in vivo models, impeding KEAP1-NRF2/HO-1 signaling in NSCLC. In xenografted mice, the NRF2 inhibitor (BM) disrupted the beneficial effects of BA and DDP, as evidenced by enhanced NSCLC xenograft tumor growth. In addition, KEAP1 knockdown reversed the protective effects of BA and DDP, showing accelerated NSCLC progression. These results explained that BA modulated the sensitivity of NSCLC to DDP in part through the KEAP1-NRF2/HO-1 pathway.

Previous reports revealed that NRF2 promoted NSCLC cell proliferation and inhibited apoptosis by enhancing autophagic activity [39]. Under certain conditions, autophagy mechanisms may overlap with cell death [40]. Ferroptosis is the newly defined iron-dependent cell death, described as the accumulation of ROS triggering iron metabolism imbalance and lipid peroxidation [41]. Ferritinophagy is a specialized type of selective autophagy, and overactivation of ferritinophagy induces iron overload to increase cellular susceptibility to ferroptosis [42]. One study demonstrated that ferritinophagy was involved in mediating the sensitivity of NSCLC cells to DDP [43]. We reported that BA and DDP favorably enhanced the activity of LC3 and increased the number of autophagosomes. In addition, BA and DDP significantly increased ROS level and lipid peroxidation and regulated the abundance of ferroptosis-related proteins SLC7A11, GPX4, SLC40A1, and transferrin, and simultaneously promoted the transition of GSH to GSSH. The combined use of BA and DDP further enhanced the effect of BA and DDP on A549 and A549/DDP cells. We concluded that BA and DDP promoted ferritinophagy in NSCLC cells, and BA enhanced DDP sensitivity. The KEAP1-NRF2 pathway is closely related to ferroptosis [44, 45]. Overexpression of KEAP1 exacerbates the degradation of NRF2, reduces the expression of HO-1, and promotes oxidative damage and ferroptosis in tumors [46]. Activation of the KEAP1/NRF2/HO-1 pathway may be triggered in ferroptosis of gastric cancer cells mediated by ferritinophagy [47]. However, studies on KEAP1/Nrf2/HO-1 targeting ferritinophagy in NSCLC are still lacking. In this study, BM reversed the accumulation of LC3 induced by BA and DDP in xenografted mice, showing that NRF2 suppressed autophagic activity in NSCLC. KEAP1 knockdown decreased the expression of LC3 II/I and transferrin and promoted the expression of SLC7A11, GPX4 and SLC40A1. KEAP1 knockdown also inhibited the transition of GSH to GSSH. These findings indicated the critical role of KEAP1-NRF2 in mediating ferritinophagy in NSCLC.

Tumor-associated macrophages are one of the relatively abundant cell types in the TME, and accumulating studies have shown that macrophages polarize to an M1 or M2 phenotype once stimulated by the environment [48]. M1-like macrophages initially functioned in the TME to suppress cancer cell growth [49]. It has been reported that the density of M1 macrophages is proportional to the survival time and prognostic effect of NSCLC patients [50]. Studies have shown that the NRF2/HO-1 pathway is involved in macrophage polarization to regulate inflammation and oxidative stress-related disorders [51, 52]. Furthermore, *SR* contributes to M1 macrophage polarization in NSCLC cells [17]. Therefore, we speculated that BA might contribute to M1 macrophage polarization in NSCLC through the KEAP1-NRF2/HO-1 pathway. In our experiments, BA and DDP increased the ratio of F4/80^+^ and CD11c^+^ and induced the accumulation of iNOS and TNF-α in cells and inhibited the activities of VEGF-A and TGF-β, indicating that BA and DDP promote M1 macrophage polarization in NSCLC. Joint administration of BA and DDP further enhanced M1 macrophage polarization in NSCLC. More importantly, BM and KEAP1 knockdown blocked the promoting effects of BA and DDP on M1 macrophage polarization.

In conclusion, BA treatment not only induced ferritinophagy in NSCLC but also promoted the polarization of M1 macrophages, ultimately improving the sensitivity of NSCLC to DDP. This process was regulated by the KEAP1-NRF2/HO-1 pathway. Our findings enhanced our understanding of BA in the treatment of NSCLC and provided a candidate therapeutic mechanism.

## Data Availability

The dataset supporting the conclusions of this article is included within the article.
